# Supplementary material for: Hot spot prediction in protein-protein interactions by an ensemble system
Source: BMC Syst Biol. 2018 Dec 31;12(Suppl 9):132. doi: 10.1186/s12918-018-0665-8 (PMC6311905; doi:10.1186/s12918-018-0665-8)
Supplement: Supplementary file 2 — All relevant MATLAB code. A simple Matlab implement of our predictor. (ZIP 19,742 kb) [file 12918_2018_665_MOESM2_ESM.zip › Additional file 2/Read ASA/ASA Property/ASEdb/1DANL.txt]

 NetSurfP - Protein Surface Accessibility and Secondary Structure Predictions

  

|  |  |  |
| --- | --- | --- |
|  |  | NetSurfP - Protein Surface Accessibility and Secondary Structure Predictions  Technical University of Denmark |

  


---

```
# For publication of results, please cite:
# A generic method for assignment of reliability scores applied to solvent accessibility predictions.
# Bent Petersen, Thomas Nordahl Petersen, Pernille Andersen, Morten Nielsen and Claus Lundegaard
# BMC Structural Biology 2009, 9:51 doi:10.1186/1472-6807-9-51
#
# Column 1: Class assignment - B for buried or E for Exposed - Threshold: 25% exposure, but not based on RSA
# Column 2: Amino acid
# Column 3: Sequence name
# Column 4: Amino acid number
# Column 5: Relative Surface Accessibility - RSA
# Column 6: Absolute Surface Accessibility
# Column 7: Z-fit score for RSA prediction
# Column 8: Probability for Alpha-Helix
# Column 9: Probability for Beta-strand
# Column 10: Probability for Coil
E A  1DANL                  1    0.773  85.185  -2.145   0.003   0.003   0.994
E N  1DANL                  2    0.530  77.651  -1.175   0.004   0.085   0.910
E A  1DANL                  3    0.471  51.849  -0.702   0.005   0.262   0.733
B F  1DANL                  4    0.178  35.745  -0.319   0.003   0.718   0.279
E L  1DANL                  5    0.477  87.320   1.500   0.001   0.900   0.099
B E  1DANL                  6    0.036   6.359  -0.870   0.001   0.900   0.099
E E  1DANL                  7    0.458  79.978   0.798   0.001   0.900   0.099
B L  1DANL                  8    0.042   7.654   0.296   0.004   0.514   0.481
E R  1DANL                  9    0.461 105.477   1.010   0.004   0.197   0.799
E P  1DANL                 10    0.706 100.238  -0.183   0.018   0.047   0.935
E G  1DANL                 11    0.401  31.582  -1.885   0.018   0.019   0.964
B S  1DANL                 12    0.263  30.847  -1.121   0.018   0.088   0.893
E L  1DANL                 13    0.463  84.702  -0.097   0.019   0.141   0.840
B E  1DANL                 14    0.233  40.775  -1.507   0.018   0.088   0.893
E R  1DANL                 15    0.660 151.071   0.851   0.018   0.047   0.935
E E  1DANL                 16    0.520  90.879   1.218   0.019   0.141   0.840
B C  1DANL                 17    0.052   7.259   0.090   0.020   0.205   0.775
E K  1DANL                 18    0.538 110.605   0.470   0.021   0.279   0.699
E E  1DANL                 19    0.676 118.185   0.334   0.020   0.205   0.775
E E  1DANL                 20    0.551  96.172  -0.312   0.018   0.088   0.893
E Q  1DANL                 21    0.533  95.122   0.465   0.019   0.141   0.840
B C  1DANL                 22    0.084  11.836  -3.632   0.021   0.279   0.699
E S  1DANL                 23    0.472  55.283  -0.233   0.021   0.279   0.699
E F  1DANL                 24    0.504 101.193  -1.737   0.018   0.088   0.893
E E  1DANL                 25    0.543  94.775  -0.719   0.018   0.019   0.964
E E  1DANL                 26    0.545  95.229  -1.235   0.018   0.019   0.964
B A  1DANL                 27    0.117  12.882  -2.056   0.005   0.045   0.951
E R  1DANL                 28    0.450 103.096  -0.116   0.004   0.197   0.799
B E  1DANL                 29    0.216  37.718  -2.223   0.005   0.262   0.733
E I  1DANL                 30    0.333  61.531  -1.389   0.004   0.197   0.799
B F  1DANL                 31    0.071  14.189  -4.265   0.004   0.420   0.576
E K  1DANL                 32    0.431  88.698  -0.021   0.004   0.616   0.381
B D  1DANL                 33    0.055   7.940  -1.979   0.021   0.451   0.528
E A  1DANL                 34    0.412  45.446  -0.535   0.019   0.141   0.840
E E  1DANL                 35    0.573 100.086  -2.261   0.018   0.088   0.893
E R  1DANL                 36    0.540 123.614  -1.532   0.018   0.047   0.935
E T  1DANL                 37    0.376  52.082  -1.690   0.018   0.088   0.893
E K  1DANL                 38    0.608 125.107  -1.742   0.019   0.141   0.840
E L  1DANL                 39    0.559 102.390  -1.818   0.004   0.138   0.858
E F  1DANL                 40    0.350  70.325  -1.376   0.005   0.262   0.733
E W  1DANL                 41    0.381  91.751  -0.328   0.005   0.262   0.733
E I  1DANL                 42    0.244  45.084  -0.686   0.021   0.451   0.528
E S  1DANL                 43    0.515  60.405  -0.013   0.021   0.451   0.528
B Y  1DANL                 44    0.124  26.563  -0.526   0.022   0.359   0.619
E S  1DANL                 45    0.601  70.496  -0.523   0.021   0.279   0.699
E D  1DANL                 46    0.479  69.038  -0.546   0.019   0.141   0.840
E G  1DANL                 47    0.339  26.695  -0.462   0.018   0.088   0.893
E D  1DANL                 48    0.462  66.502  -1.146   0.018   0.047   0.935
E Q  1DANL                 49    0.384  68.547   0.558   0.018   0.088   0.893
B C  1DANL                 50    0.022   3.117  -1.021   0.020   0.205   0.775
E A  1DANL                 51    0.427  47.088  -0.029   0.020   0.205   0.775
E S  1DANL                 52    0.645  75.606  -1.935   0.018   0.088   0.893
E S  1DANL                 53    0.390  45.731  -0.778   0.005   0.015   0.979
E P  1DANL                 54    0.435  61.726  -1.691   0.005   0.015   0.979
B C  1DANL                 55    0.088  12.313  -2.344   0.005   0.015   0.979
E Q  1DANL                 56    0.432  77.137  -0.713   0.005   0.015   0.979
B N  1DANL                 57    0.328  47.961  -1.768   0.005   0.015   0.979
E G  1DANL                 58    0.541  42.608  -1.071   0.005   0.015   0.979
B G  1DANL                 59    0.064   5.005  -2.923   0.004   0.138   0.858
E S  1DANL                 60    0.372  43.552   1.581   0.003   0.718   0.279
B C  1DANL                 61    0.015   2.176   0.890   0.002   0.816   0.182
E K  1DANL                 62    0.357  73.394   0.937   0.021   0.756   0.223
E D  1DANL                 63    0.316  45.550  -0.441   0.004   0.420   0.576
E Q  1DANL                 64    0.419  74.905   0.342   0.005   0.045   0.951
E L  1DANL                 65    0.648 118.594  -1.930   0.005   0.015   0.979
E Q  1DANL                 66    0.639 114.161  -1.233   0.005   0.015   0.979
E S  1DANL                 67    0.346  40.551  -0.087   0.004   0.197   0.799
B Y  1DANL                 68    0.122  26.136   0.194   0.003   0.718   0.279
E I  1DANL                 69    0.451  83.435   1.700   0.001   0.900   0.099
B C  1DANL                 70    0.017   2.443   0.377   0.001   0.900   0.099
E F  1DANL                 71    0.427  85.699   1.336   0.001   0.900   0.099
B C  1DANL                 72    0.025   3.524   0.874   0.021   0.451   0.528
E L  1DANL                 73    0.499  91.312   0.890   0.018   0.088   0.893
E P  1DANL                 74    0.715 101.473   0.272   0.018   0.047   0.935
E A  1DANL                 75    0.427  47.044  -1.922   0.018   0.019   0.964
B F  1DANL                 76    0.146  29.242  -0.493   0.018   0.047   0.935
E E  1DANL                 77    0.567  99.020   0.057   0.019   0.141   0.840
B G  1DANL                 78    0.204  16.023  -1.235   0.018   0.088   0.893
E R  1DANL                 79    0.638 146.102   1.018   0.018   0.088   0.893
E N  1DANL                 80    0.554  81.135   0.556   0.018   0.047   0.935
B C  1DANL                 81    0.146  20.428  -0.553   0.018   0.088   0.893
E E  1DANL                 82    0.602 105.204  -0.091   0.019   0.141   0.840
E T  1DANL                 83    0.492  68.185   0.707   0.022   0.359   0.619
E H  1DANL                 84    0.561 102.119   1.489   0.022   0.359   0.619
B K  1DANL                 85    0.117  24.046   1.040   0.066   0.296   0.638
E D  1DANL                 86    0.657  94.674   0.181   0.066   0.296   0.638
E D  1DANL                 87    0.426  61.387   0.576   0.066   0.296   0.638
B Q  1DANL                 88    0.127  22.664  -0.723   0.056   0.142   0.802
E L  1DANL                 89    0.503  92.026  -0.623   0.056   0.142   0.802
E I  1DANL                 90    0.629 116.309  -0.518   0.018   0.088   0.893
B C  1DANL                 91    0.255  35.858  -0.960   0.018   0.047   0.935
E V  1DANL                 92    0.405  62.310  -0.871   0.018   0.047   0.935
B N  1DANL                 93    0.179  26.220  -2.240   0.018   0.019   0.964
E E  1DANL                 94    0.572  99.998  -0.628   0.018   0.019   0.964
E N  1DANL                 95    0.444  65.031  -2.631   0.018   0.019   0.964
E G  1DANL                 96    0.402  31.598  -2.292   0.005   0.015   0.979
B G  1DANL                 97    0.261  20.564  -1.963   0.005   0.015   0.979
B C  1DANL                 98    0.156  21.846  -0.747   0.004   0.197   0.799
E E  1DANL                 99    0.339  59.293  -0.689   0.004   0.420   0.576
B Q  1DANL                100    0.216  38.631  -0.927   0.004   0.616   0.381
E Y  1DANL                101    0.341  72.872   0.353   0.002   0.816   0.182
B C  1DANL                102    0.043   6.093  -0.993   0.021   0.756   0.223
E S  1DANL                103    0.330  38.676   0.229   0.018   0.846   0.136
B D  1DANL                104    0.279  40.218  -1.173   0.004   0.420   0.576
E H  1DANL                105    0.391  71.105  -0.621   0.005   0.045   0.951
E T  1DANL                106    0.632  87.589  -1.661   0.005   0.015   0.979
E G  1DANL                107    0.697  54.846  -1.684   0.003   0.003   0.994
E T  1DANL                108    0.397  55.092  -0.798   0.005   0.045   0.951
E K  1DANL                109    0.456  93.779   0.121   0.005   0.262   0.733
B R  1DANL                110    0.097  22.305   0.647   0.003   0.718   0.279
E S  1DANL                111    0.439  51.463   1.454   0.001   0.900   0.099
B C  1DANL                112    0.017   2.443   0.495   0.001   0.900   0.099
E R  1DANL                113    0.419  95.997   0.989   0.002   0.816   0.182
B C  1DANL                114    0.026   3.594   0.897   0.022   0.359   0.619
E H  1DANL                115    0.492  89.477   0.928   0.018   0.047   0.935
E E  1DANL                116    0.697 121.836   0.158   0.018   0.019   0.964
E G  1DANL                117    0.385  30.268  -1.942   0.018   0.019   0.964
B Y  1DANL                118    0.130  27.696  -0.354   0.019   0.141   0.840
E S  1DANL                119    0.523  61.342   0.118   0.021   0.279   0.699
B L  1DANL                120    0.222  40.703  -1.400   0.020   0.205   0.775
E L  1DANL                121    0.554 101.437   0.520   0.018   0.088   0.893
E A  1DANL                122    0.634  69.900   0.301   0.018   0.047   0.935
E D  1DANL                123    0.338  48.677  -0.279   0.018   0.019   0.964
E G  1DANL                124    0.641  50.415  -0.534   0.018   0.047   0.935
E V  1DANL                125    0.471  72.423   0.949   0.020   0.205   0.775
E S  1DANL                126    0.536  62.831   1.563   0.021   0.279   0.699
B C  1DANL                127    0.059   8.354   0.367   0.022   0.359   0.619
E T  1DANL                128    0.608  84.343   0.700   0.022   0.359   0.619
E P  1DANL                129    0.504  71.574  -0.030   0.066   0.296   0.638
E T  1DANL                130    0.284  39.446   0.013   0.056   0.142   0.802
E V  1DANL                131    0.493  75.836  -1.012   0.052   0.084   0.864
E E  1DANL                132    0.576 100.697  -0.225   0.052   0.084   0.864
B Y  1DANL                133    0.258  55.177  -0.771   0.018   0.047   0.935
E P  1DANL                134    0.494  70.155  -0.678   0.018   0.047   0.935
B C  1DANL                135    0.173  24.261  -2.965   0.018   0.088   0.893
E G  1DANL                136    0.385  30.284  -1.385   0.018   0.047   0.935
E K  1DANL                137    0.677 139.177  -1.109   0.018   0.019   0.964
E I  1DANL                138    0.365  67.543  -1.136   0.018   0.019   0.964
E P  1DANL                139    0.439  62.322  -0.695   0.005   0.015   0.979
B I  1DANL                140    0.043   7.936  -1.272   0.005   0.045   0.951
E L  1DANL                141    0.453  82.908  -0.005   0.018   0.047   0.935
E E  1DANL                142    0.416  72.658  -1.092   0.005   0.015   0.979
E K  1DANL                143    0.595 122.330  -1.493   0.003   0.003   0.994
B R  1DANL                144    0.138  31.579  -1.150   0.004   0.085   0.910
E N  1DANL                145    0.489  71.516   1.287   0.003   0.718   0.279
B A  1DANL                146    0.024   2.667  -0.287   0.002   0.816   0.182
E S  1DANL                147    0.440  51.580   0.344   0.003   0.718   0.279
E K  1DANL                148    0.270  55.580   0.226   0.005   0.336   0.660
E P  1DANL                149    0.482  68.367   0.421   0.004   0.138   0.858
E Q  1DANL                150    0.731 130.574  -0.160   0.018   0.047   0.935
E G  1DANL                151    0.548  43.120  -1.919   0.018   0.019   0.964
E R  1DANL                152    0.553 126.706  -0.335   0.003   0.003   0.994

Explain the output.  Go back.
```

---
